# Supplementary material for: Immunogenicity of a spike protein subunit-based COVID-19 vaccine with broad protection against various SARS-CoV-2 variants in animal studies
Source: PLoS One. 2023 Mar 24;18(3):e0283473. doi: 10.1371/journal.pone.0283473 (PMC10038307; doi:10.1371/journal.pone.0283473)
Supplement: S1 Fig — The delta S protein (10 μg) combined with or without adjuvant candidates of aluminum hydroxide plus CpG 1018 (50 μg plus 10 μg, triangle), AB801 (5 or 10 μg, square), or AB801-ISCOM (5 or 10 μg, circle, grey bars) were immunized to BALB/c mice on day 0 and day 14. The anti-S protein IgG titer of (A) alpha, (B) beta, and (C) gamma strains in immunized mouse serum collected on day 28. Data were analyzed using one-way ANOVA. (PDF) [file pone.0283473.s001.pdf]

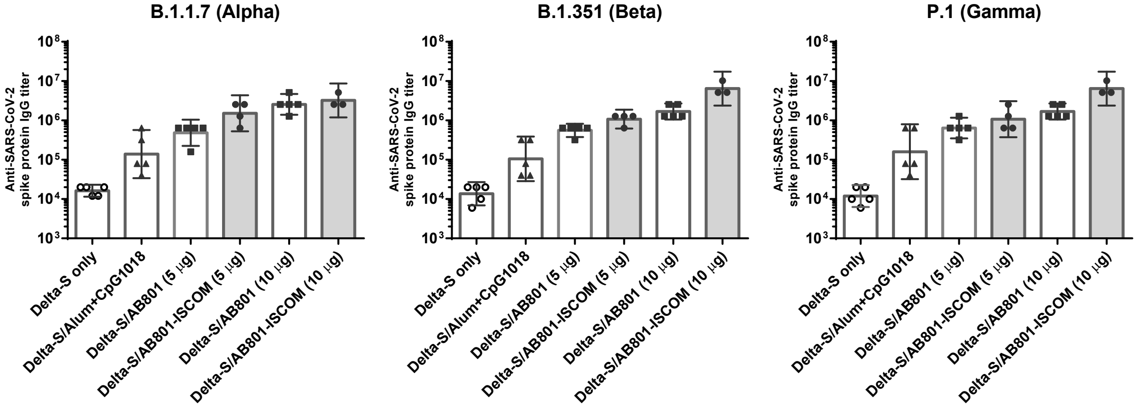

Fig S1. Serum IgG titers of BCVax immunized BALB/c mice.

The delta S protein (10 μg) combined with or without adjuvant candidates of aluminum hydroxide plus CpG 1018 (50 μg plus 10 μg, triangle), AB801 (5 or 10 μg, square), or AB801-ISCOM (5 or 10 μg, circle, grey bars) were immunized to BALB/c mice on day 0 and day 14. The anti-S protein IgG titer of (A) alpha, (B) beta, and (C) gamma strains in immunized mouse serum collected on day 28. Data were analyzed using one-way ANOVA.
