# Supplementary figures and images for: Immunogenicity of a spike protein subunit-based COVID-19 vaccine with broad protection against various SARS-CoV-2 variants in animal studies
Source: PLoS One. 2023 Mar 24;18(3):e0283473. doi: 10.1371/journal.pone.0283473 (PMC10038307; doi:10.1371/journal.pone.0283473)

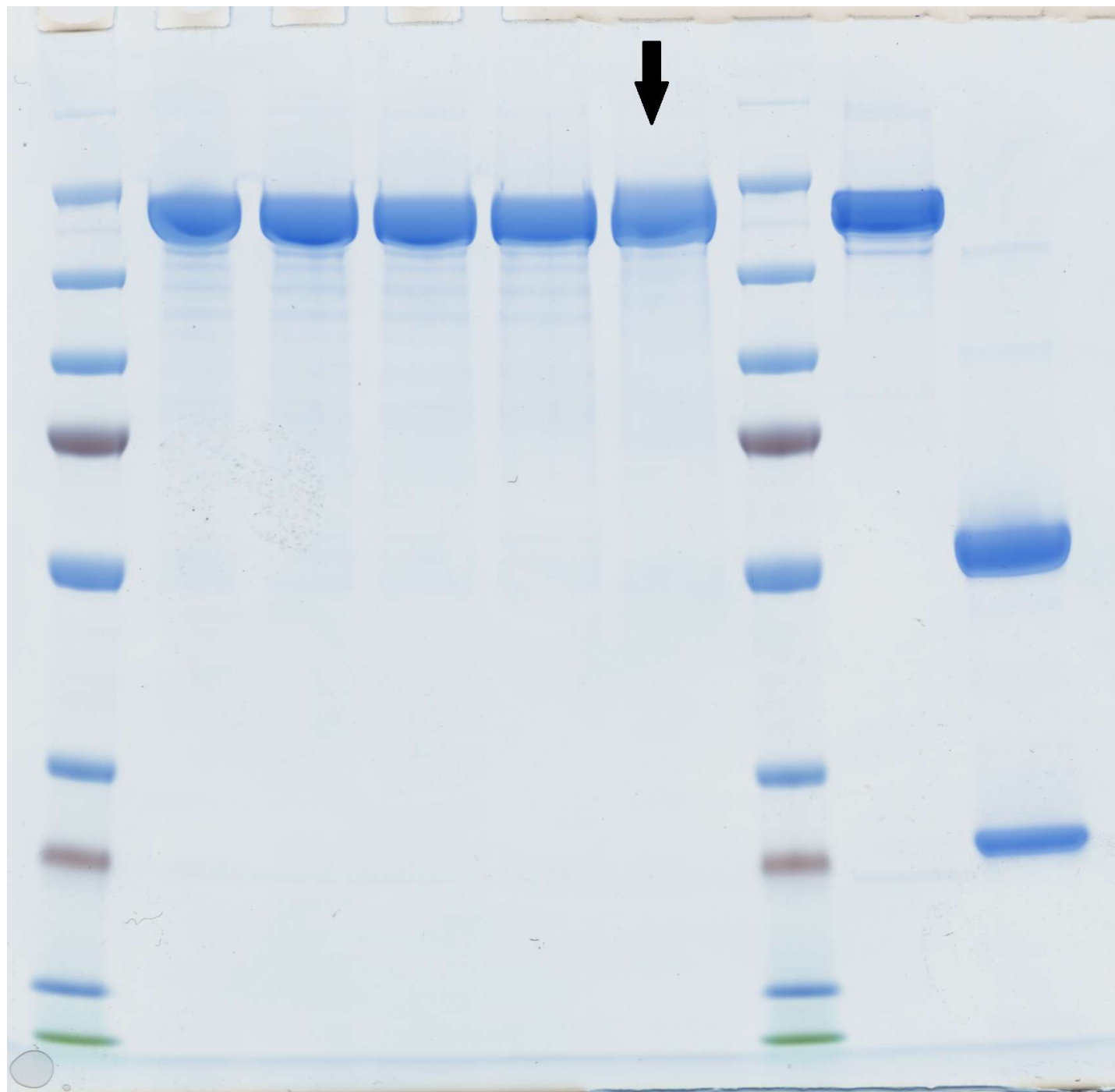

Supplement: S1 File — (PDF) [file pone.0283473.s002.pdf]
