# Supplementary material for: Immunogenicity of a spike protein subunit-based COVID-19 vaccine with broad protection against various SARS-CoV-2 variants in animal studies
Source: PLoS One. 2023 Mar 24;18(3):e0283473. doi: 10.1371/journal.pone.0283473 (PMC10038307; doi:10.1371/journal.pone.0283473)
Supplement: S2 File — (PDF) [file pone.0283473.s003.pdf]

Mouse serum IgG titer at Day 28 (Fig 3B)

Group Name	Mouse #	Wild type	Delta type	Omicron type (BA.1)	Omicron type (BA.2)	Omicron type (BA.5)
Delta-S only	1	40000	40000	20000	12000	24000
	2	10000	10000	3000	3000	6000
	3	40000	40000	20000	6000	48000
	4	10000	10000	3000	3000	12000
	5	40000	40000	20000	6000	48000
Delta-S/Alum+CpG 1018	1	640000	320000	320000	160000	160000
	2	80000	160000	80000	80000	80000
	3	1280000	640000	80000	160000	160000
	4	80000	80000	40000	40000	40000
	5	80000	80000	80000	40000	40000
Delta-S/AB801 (5 µg)	1	320000	640000	320000	320000	320000
	2	1280000	1280000	320000	160000	160000
	3	640000	640000	640000	160000	160000
	4	640000	640000	640000	160000	160000
	5	640000	640000	1280000	320000	320000
Delta-S/AB801-ISCOM (5 µg)	1	1280000	2560000	640000	1280000	1280000
	2	2560000	5120000	1280000	320000	320000
	3	1280000	1280000	640000	320000	320000
	4	2560000	2560000	1280000	640000	640000
Delta-S/AB801 (10 µg)	1	2560000	1280000	1280000	640000	5120000
	2	2560000	1280000	1280000	640000	5120000
	3	2560000	2560000	1280000	640000	2560000
	4	1280000	1280000	1280000	1280000	2560000
	5	5120000	2560000	1280000	1280000	5120000
Delta-S/AB801-ISCOM (10 µg)	1	5120000	10240000	2560000	2560000	5120000
	2	10240000	10240000	5120000	2560000	5120000
	3	5120000	5120000	2560000	1280000	5120000

Pseudovirus neutralization antibody titer of Day 28 serum (Fig 3C)

Group Name	Mouse #	D614G	Alpha type (B.1.1.7)	Beta type (507Y.V2)	Gamma type (P.1)	Delta type (B.1.617.2)	Omicron type (BA.1)	Omicron type (BA.2)	Omicron type (BA.4/BA.5)
Delta-S only	1	<500	<500	<500	<500	<500	<500	<500	<500
	2	<500	<500	<500	<500	<500	<500	<500	<500
	3	<500	<500	<500	<500	<500	<500	<500	<500
	4	<500	<500	<500	<500	<500	<500	<500	<500
	5	<500	<500	<500	<500	<500	<500	<500	<500
Delta-S/Alum+CpG 1018	1	<500	690	<500	517	<500	<500	<500	<500
	2	<500	<500	<500	<500	<500	<500	<500	<500
	3	2499	3290	1008	2253	2588	<500	<500	<500
	4	<500	738	<500	<500	<500	<500	<500	<500
	5	<500	<500	<500	<500	<500	<500	<500	<500
Delta-S/AB801 (5 µg)	1	4067	722	<500	635	2116	<500	<500	<500
	2	6834	2069	1946	3781	9663	<500	<500	<500
	3	8186	2942	2078	3703	7532	<500	<500	<500
	4	2502	2382	<500	1728	3687	<500	<500	<500
	5	2675	1653	1271	2425	12739	<500	<500	<500
Delta-S/AB801-ISCOM (5 µg)	1	2388	5387	876	2717	4657	<500	<500	<500
	2	6227	5653	1703	5828	9380	<500	<500	<500
	3	687	2912	698	3062	1165	<500	<500	<500
	4	2897	16609	1944	16102	5872	1277	819	<500
Delta-S/AB801 (10 µg)	1	8724	17678	8074	12619	3405	500	500	<500
	2	5267	9464	1261	7645	3812	1727	2213	<500
	3	38082	32959	9561	20766	25902	10597	12482	5308
	4	1756	3993	1472	4020	18024	31731	86728	2464
	5	11464	16637	1037	20208	9539	500	500	1027
Delta-S/AB801-ISCOM (10 µg)	1	16316	10033	6183	11844	33399	33500	46173	<500
	2	19108	64000	6910	42482	20029	1558	2140	<500
	3	28084	16529	4128	12381	32767	8300	11292	2865

Pseudovirus neutralization antibody titer of Day 28 serum (Fig 3D)

Group Name	Mouse #	D614G	Alpha type (B.1.1.7)	Beta type (507Y.V2)	Gamma type (P.1)	Delta type (B.1.617.2)	Omicron type (BA.1)	Omicron type (BA.2)	Omicron type (BA.4/BA.5)
Delta-S only	1	<500	<500	<500	<500	<500	<500	<500	<500
	2	<500	<500	<500	<500	<500	<500	<500	<500
	3	<500	<500	<500	<500	<500	<500	<500	<500
	4	<500	<500	<500	<500	<500	<500	<500	<500
	5	<500	<500	<500	<500	<500	<500	<500	<500
Delta-S/AB801-ISCOM (5 μg)	1	2388	5387	876	2717	4657	<500	<500	<500
	2	6227	5653	1703	5828	9380	<500	<500	<500
	3	687	2912	698	3062	1165	<500	<500	<500
	4	2897	16609	1944	16102	5872	1277	819	<500
Delta-S/AB801-ISCOM (10 μg)	1	16316	10033	6183	11844	33399	33500	46173	<500
	2	19108	64000	6910	42482	20029	1558	2140	<500
	3	28084	16529	4128	12381	32767	8300	11292	2865

T cell responses in BCVax immunized BALB/c mice (Fig 4A, 4B)

Fig.04 A and B: Flow Cytometer Analysis of CD4 and CD8 T cell populations

Test Sample			$CD4^+$ T cells ($CD4^+$)			IFN-g^+ cells ($CD4^+$IFN-g^+)			IL-4^+ cells ($CD4^+$IL-4^+)			$CD8^+$ T cells ($CD8^+$)			IFN-g^+ cells ($CD8^+$IFN-g^+)			Granzyme B^+ cells ($CD8^+$GrB^+)		
			Total%	cells Gated %	cell number (x10e5)	Total%	$CD4^+$ Gated %	cell number (x10e4)	Total%	$CD4^+$ Gated %	cell number (x10e4)	Total%	cells Gated %	cell number (x10e5)	Total%	$CD8^+$ Gated %	cell number (x10e4)	Total%	$CD8^+$ Gated %	cell number (x10e4)
Isotype control			0.06	0.17		0.04	0.32		0.01	0.08		0.04	0.11		0.00	0.08		0.01	0.21	
DS2P	23	Peptide pool mix 2 μg/ml	9.99	34.00	2.00	0.02	0.22	0.04	0.01	0.12	0.02	3.94	13.39	0.79	0.01	0.20	0.02	0.00	0.00	0.00
	27		11.00	38.92	2.20	0.02	0.20	0.04	0.01	0.13	0.02	4.64	16.43	0.93	0.01	0.30	0.02	0.01	0.17	0.02
	34		11.12	33.99	2.22	0.02	0.20	0.04	0.02	0.20	0.04	4.82	14.73	0.96	0.00	0.04	0.00	0.00	0.04	0.00
	40		11.32	34.53	2.26	0.02	0.21	0.04	0.02	0.18	0.04	4.44	13.55	0.89	0.01	0.18	0.02	0.00	0.05	0.00
	42		12.48	37.44	2.50	0.03	0.21	0.06	0.01	0.06	0.02	5.14	15.41	1.03	0.01	0.16	0.02	0.00	0.00	0.00
		Mean					0.21			0.14						0.18			0.05	
		STD					0.01			0.05						0.09			0.07	
DS2P/AB801 (10 μg)	2	Peptide pool mix 2 μg/ml	12.86	35.12	2.57	0.10	0.78	0.20	0.02	0.12	0.04	5.47	14.95	1.09	0.02	0.37	0.04	0.00	0.04	0.00
	19		11.38	33.75	2.28	0.09	0.77	0.18	0.02	0.19	0.04	4.71	13.97	0.94	0.03	0.72	0.06	0.00	0.00	0.00
	20		9.39	32.00	1.88	0.04	0.47	0.08	0.01	0.09	0.02	3.42	11.64	0.68	0.02	0.64	0.04	0.00	0.00	0.00
	21		10.47	31.50	2.09	0.07	0.67	0.14	0.02	0.15	0.04	4.75	14.28	0.95	0.03	0.72	0.06	0.00	0.00	0.00
	26		12.51	37.01	2.50	0.05	0.38	0.10	0.01	0.05	0.02	5.09	15.05	1.02	0.03	0.63	0.06	0.00	0.00	0.00
		Mean					0.61			0.12						0.62			0.01	
		STD					0.18			0.05						0.14			0.02	
DS2P/AB801-ISCOM	12	Peptide pool mix 2 μg/ml	13.85	37.63	2.77	0.12	0.88	0.24	0.03	0.20	0.06	5.87	15.93	1.17	0.13	2.15	0.26	0.01	0.10	0.02
	16		12.60	36.53	2.52	0.13	1.05	0.26	0.02	0.13	0.04	5.07	14.70	1.01	0.05	0.99	0.10	0.01	0.12	0.02
	39		12.36	34.99	2.47	0.09	0.70	0.18	0.01	0.08	0.02	5.26	14.91	1.05	0.06	1.18	0.12	0.00	0.08	0.00
		Mean					0.88			0.14						1.44			0.10	
		STD					0.18			0.06						0.62			0.02	
DS2P/Alum+CpG1018	9	Peptide pool mix 2 μg/ml	12.81	32.92	2.56	0.04	0.34	0.08	0.01	0.09	0.02	4.57	11.74	0.91	0.01	0.26	0.02	0.00	0.00	0.00
	24		14.77	37.05	2.95	0.07	0.46	0.14	0.02	0.15	0.04	5.41	13.58	1.08	0.02	0.37	0.04	0.00	0.00	0.00
	30		12.40	32.46	2.48	0.02	0.19	0.04	0.01	0.06	0.02	5.35	14.02	1.07	0.02	0.30	0.04	0.01	0.11	0.02
	31		10.62	30.05	2.12	0.03	0.26	0.06	0.01	0.13	0.02	4.21	11.92	0.84	0.00	0.05	0.00	0.00	0.00	0.00
	38		10.71	30.84	2.14	0.04	0.39	0.08	0.01	0.07	0.02	3.77	10.86	0.75	0.01	0.37	0.02	0.00	0.05	0.00
		Mean					0.33			0.10						0.27			0.03	
		STD					0.11			0.04						0.13			0.05	
DS2P/AB801 (5 μg)	1	Peptide pool mix 2 μg/ml	12.30	33.18	2.46	0.16	1.32	0.32	0.02	0.16	0.04	5.25	14.17	1.05	0.07	1.26	0.14	0.01	0.11	0.02
	8		13.27	37.05	2.65	0.03	0.23	0.06	0.01	0.06	0.02	5.07	14.17	1.01	0.03	0.67	0.06	0.00	0.04	0.00
	13		12.60	34.55	2.52	0.05	0.40	0.10	0.01	0.05	0.02	5.24	14.38	1.05	0.02	0.46	0.04	0.00	0.04	0.00
	14		11.18	30.94	2.24	0.05	0.45	0.10	0.02	0.16	0.04	4.85	13.43	0.97	0.01	0.21	0.02	0.00	0.00	0.00
	32		14.64	39.03	2.93	0.21	1.46	0.42	0.02	0.14	0.04	4.25	11.32	0.85	0.04	0.99	0.08	0.00	0.00	0.00
		Mean					0.77			0.11						0.72			0.04	
		STD					0.57			0.05						0.42			0.04	
DS2P/AB801-ISCOM (5 μg)	3	Peptide pool mix 2 μg/ml	10.26	33.49	2.05	0.01	0.14	0.02	0.01	0.06	0.02	4.34	14.18	0.87	0.07	1.66	0.14	0.00	0.09	0.00
	7		14.52	37.70	2.90	0.05	0.37	0.10	0.01	0.07	0.02	6.55	17.00	1.31	0.09	1.37	0.18	0.00	0.03	0.00
	10		14.59	37.70	2.92	0.07	0.49	0.14	0.00	0.03	0.00	6.94	17.92	1.39	0.06	0.92	0.12	0.01	0.09	0.02
	11		12.87	33.16	2.57	0.06	0.47	0.12	0.02	0.14	0.04	5.47	14.08	1.09	0.04	0.73	0.08	0.01	0.15	0.02
		Mean					0.37			0.08						1.17			0.09	
		STD					0.16			0.05						0.42			0.05	

T cell responses in BCVax immunized BALB/c mice (Fig 4C)

Fig.04 C and D:ELISPOT								
			IL-2	IFN-γ	IL-4	IFN-γ/IL-4 Ratio	Mean	STD
DS2P	23	Peptide pool mix 2 μg/ml	29.50	56.00	64.00	0.88	0.78	0.14
	27		21.00	47.50	51.00	0.93		
	34		29.50	46.00	55.50	0.83		
	40		29.50	59.00	95.00	0.62		
	42		33.00	57.00	89.00	0.64		
DS2P/AB801 (10 μg)	0	Peptide pool mix 2 μg/ml	284.00	345.00	94.00	3.67	2.69	1.09
	19		186.50	222.50	61.00	3.65		
	20		168.00	226.50	79.50	2.85		
	21		43.00	73.50	70.00	1.05		
	26		115.50	177.00	78.50	2.25		
DS2P/AB801-ISCOM	12	Peptide pool mix 2 μg/ml	333.50	439.00	78.00	5.63	4.22	1.30
	16		222.50	303.00	99.00	3.06		
	39		327.50	438.50	110.50	3.97		
DS2P/Alum+CpG1018	9	Peptide pool mix 2 μg/ml	60.00	75.00	60.00	1.25	1.58	0.61
	24		28.50	59.50	48.00	1.24		
	30		38.00	59.00	23.00	2.57		
	31		33.00	37.00	34.00	1.09		
	38		32.00	52.50	30.00	1.75		
DS2P/AB801 (5 μg)	1	Peptide pool mix 2 μg/ml	63.50	75.50	42.50	1.78	4.01	4.82
	8		74.00	195.50	15.50	12.61		
	13		84.50	125.00	68.00	1.84		
	14		89.50	90.50	41.50	2.18		
	32		162.00	126.50	78.00	1.62		
DS2P/AB801-ISCOM (5 μg)	3	Peptide pool mix 2 μg/ml	81.00	179.50	41.50	4.33	4.21	1.59
	7		145.00	273.50	43.00	6.36		
	10		144.00	223.50	63.50	3.52		
	11		134.00	108.00	41.00	2.63		

Mouse serum IgG titer of Day 84 (Fig 5B)

Group#	Mouse #	Delta type	Omicron (BA.2) type	Omicron (BA.5) type
Delta-S/ AB801-ISCOM (2 µg)	1	409600	102400	102400
	2	409600	102400	102400
	3	409600	204800	204800
Delta-S/ AB801-ISCOM (5 µg)	1	1638400	409600	819200
	2	819200	204800	204800
	3	819200	204800	204800
Delta-S/ AB801-ISCOM (7.5 µg)	1	3276800	819200	1638400
	2	1638400	409600	1638400
	3	1638400	409600	819200
Boost Delta-S/ AB801-ISCOM (7.5 µg)	1	409600	13107200	3276800
	2	409600	204800	52428800
	3	6553600	6553600	6553600
	4	13107200	13107200	6553600

Geometric mean of Mouse serum IgG titer of Day 84 (Fig 5C)

Type	Name	Week					
		0	2	4	8	10	12
Delta type	Delta-S/ AB801-ISCOM (7.5 µg)	50	56529	3360094	2600798	1638400	2064255
Delta type	Boost Delta-S/ AB801-ISCOM (7.5 µg)	50	38802	1552094	3276800	9268190	1948397
Omicron (BA.2) type	Delta-S/ AB801-ISCOM (7.5 µg)	50	25600	1024000	2064255	1300399	516064
Omicron (BA.2) type	Boost Delta-S/ AB801-ISCOM (7.5 µg)	50	19401	891444	688862	2317048	3896794
Omicron (BA.5) type	Delta-S/ AB801-ISCOM (7.5 µg)	50	56529	1680047	1300399	1032127	1300399
Omicron (BA.5) type	Boost Delta-S/ AB801-ISCOM (7.5 µg)	50	33779	3565775	1377725	6553600	9268190

Pseudovirus neutralization antibody titer of Day 84 serum (Fig 5D)

Group Name	Mouse #	Delta type (B.1.617.2)	Omicron type (BA.2)	Omicron type (BA.4/BA.5)
Delta-S/ AB801-ISCOM (2 µg)	1	1782	313	<250
	2	541	<250	<250
	3	532	<250	<250
Delta-S/ AB801-ISCOM (5 µg)	1	911	584	502
	2	1296	574	398
	3	2756	<250	<250
Delta-S/ AB801-ISCOM (7.5 µg)	1	8082	1320	193
	2	1989	1892	1301
	3	14899	<250	104
Boost Delta-S/ AB801-ISCOM (7.5 µg)	1	2246.	4465	541
	2	54134	6982	6445
	3	9741	3804	2653
	4	55907	11632	17130

Evaluation of the immunogenicity of BCVax candidates in BALB/c mice with booster injection (Fig 5E)

Fig.05 E:Flow Cytometer Analysis of CD4 and CD8 T cell populations

Test Sample			$CD8^+$ T cells			Granzyme B^+ cells			IFN-g^+ cells		
			Total%	cells Gated %	cell number (x10e5)	Total%	$CD8^+$ Gated %	cell number (x10e4)	Total%	$CD8^+$ Gated %	cell number (x10e4)
Isotype control			0.04	0.08	0.01	0.07	1.23	0.14	0.00	0.00	0.00
DS2P	6	Peptide pool mix 2 µg/ml	3.66	11.18	0.73	0.01	0.16	0.02	0.01	0.02	0.00
	17		4.68	12.45	0.94	0.00	0.09	0.00	0.01	0.01	0.00
	36		3.57	10.22	0.71	0.00	0.11	0.00	0.00	0.03	0.00
	39		4.21	11.58	0.84	0.01	0.19	0.02	0.01	0.03	0.00
	52		4.80	12.37	0.96	0.01	0.17	0.02	0.01	0.01	0.00
		Mean					0.14			0.02	
		STD					0.04			0.01	
DS2P/AB801-ISCOM (7.5 µg)	26	Peptide pool mix 2 µg/ml	6.00	12.97	1.20	0.04	0.63	0.08	0.00	0.07	0.00
	35		4.47	10.43	0.89	0.03	0.63	0.06	0.01	0.18	0.02
	40		4.57	11.20	0.91	0.03	0.70	0.06	0.01	0.22	0.02
		Mean					0.65			0.16	
		STD					0.04			0.08	
DS2P/AB801-ISCOM (5 µg)	1	Peptide pool mix 2 µg/ml	6.80	13.08	1.00	0.04	0.65	0.07	0.02	0.24	0.01
	24		5.87	12.19	0.10	0.02	0.34	0.01	0.01	0.17	0.01
	30		6.79	14.03	1.36	0.03	0.47	0.06	0.00	0.06	0.00
		Mean	6.49	13.10	0.82	0.03	0.49	0.04	0.01	0.16	0.01
		STD	0.53	0.92	0.65	0.01	0.16	0.03	0.01	0.09	0.01
DS2P/AB801-ISCOM (2 µg)	10	Peptide pool mix 2 µg/ml	6.06	11.71	1.21	0.03	0.46	0.06	0.01	0.13	0.02
	27		5.39	10.84	1.08	0.01	0.22	0.02	0.00	0.04	0.00
	50		4.94	10.19	1.22	0.01	0.28	0.05	0.00	0.00	0.01
		Mean	5.46	10.91	1.17	0.02	0.32	0.04	0.00	0.06	0.01
		STD	0.56	0.76	0.08	0.01	0.12	0.02	0.01	0.07	0.01
Boost DS2P/AB801-ISCOM (7.5 µg)	9	Peptide pool mix 2 µg/ml	9.26	15.96	1.85	0.11	1.19	0.22	0.06	0.60	0.12
	11		8.15	16.53	1.63	0.10	1.28	0.20	0.03	0.34	0.06
	43		6.67	14.30	1.33	0.08	1.20	0.16	0.02	0.30	0.04
	46		7.94	15.44	1.59	0.09	1.16	0.18	0.07	0.91	0.14
		Mean					1.21			0.54	
		STD					0.05			0.28	
