# Supplementary material for: Immunogenicity of a spike protein subunit-based COVID-19 vaccine with broad protection against various SARS-CoV-2 variants in animal studies
Source: PLoS One. 2023 Mar 24;18(3):e0283473. doi: 10.1371/journal.pone.0283473 (PMC10038307; doi:10.1371/journal.pone.0283473)
Supplement: S3 File — (PDF) [file pone.0283473.s004.pdf]

Determination of Mouse Anti-SARS CoV-2 spike protein IgG antibody in Serum by titer ELISA

1. Reagent and buffer preparation:
    a. 1X Carbonate-Bicarbonate buffer: Dissolve 1 cap of Carbonate-Bicarbonate powder (Sigma, C3041) with 100 mL of DI water to make 1X Carbonate-Bicarbonate Buffer.
    b. Coating solution: Add 10 µL SARS-CoV-2 spike protein (Delta type, AcroBiosystem, SPN-C52He) (0.6 mg/mL) into 5.99 mL of carbonate-bicarbonate buffer and gently mix upside down (Do not vortex)
    c. 1X PBST (0.05% Tween 20) buffer: Add 2.5 mL Tween-20 into 500 mL of 10X PBS then mix with 4.5 L DI water to make 1X PBST.
    d. 1X PBS buffer: Mix 100 mL of 10X PBS with 900 mL DI water then filtered by 0.22 µm filter membrane.
    e. 1% BSA blocking buffer contain 0.05% Tween-20: Mix 2 mL Blocker BSA (10X), 10 µL Tween-20 and 17.99 mL of 1X PBS to make 1% BSA blocking buffer.
    f. QC serum:
        i. Positive control (PC) serum stock preparation: Add mouse anti-spike protein IgG antibody into Normal BABL/c female mouse sera to make 24 µg/mL of high positive control (HPC) stock serum and 0.3 µg/mL of low positive control (LPC) stock serum.
        ii. PC serum sample and NC (negative control) sample dilution: add 5 µL of PC sample (HPC & LPC) or negative control sample (normal serum) to 495 µL of blocking buffer in an eppendorf and mix well (100-fold dilution).
    g. Test Serum sample dilution: Add 2 µL of serum sample to 78 µL of blocking buffer on 96 well plate and mix well to make 40X diluted sample. Then transfer 10 µL of 40X diluted sample to 90 µL of blocking buffer on 96 well plate and mix well to make 400X dilution. Then transfer 25 µL of 400X diluted sample to 175 µL of blocking buffer on 96 well plate and mix well to make 3200X diluted sample which the first serial dilution sample. Make 3-fold serial dilution from 3200-fold to 6998400-fold dilution by transfer 80 µL sample of the first well into the second well contain 160 µL blocking buffer and mix well, move 80 µL sample of that into the next, and so on.
    h. Goat anti Mouse IgG-HRP solution: Add 2 µL of goat anti mouse IgG-HRP antibody to 198 µL of blocking buffer to make 100X diluted sample. Then add 30 µL 100X diluted sample into 5.97 mL of blocking buffer to make 20000X diluted goat anti mouse IgG-HRP solution.

2. Plate coating:
    a. Apply 50 µL of coating solution into 96 well plate each well and seal membrane on the plate. The plate is put at 2~8°C overnight.
    b. Take plate out and warm up it to room temperature and wash by 200 µL PBST for three times.

3. ELISA assay:
    a. Add 50 µL of blocking buffer into 96 well plate and incubate the plate at 25°C for 30 mins.
    b. Aspirate the wells and wash with ELISA washer (BioTek) using 200 µL PBST for three times.
    c. Dispense 50 µL of the NC (negative control), HPC (high positive control), LPC (low positive control) samples (from Step 1.f.ii) and Serum Samples into the designated wells. Incubate the wells at 25°C for 1 hour.
    d. Aspirate the wells and wash with 200 µL PBST for three times.
    e. Dispense 50 µL of goat anti-Mouse IgG-HRP solution into wells. Incubate the wells at 25°C for 1 hour.
    f. Aspirate the wells and wash with 200 µL PBST for three times.
    g. Dispense 100 µL of TMB reagent into wells. Incubate the wells at 25°C for 15 minutes.

h. Stop the reaction by adding 100 µL of Stop Solution (1N HCl) into wells. Gently mix for 10 seconds, ensuring a complete color change from blue to yellow.
i. Read plate at 450 nm on a microplate reader within 15 minutes.

4. Data analysis:
   a. Identify outlier for NC and PC by GraphPad Prism. Outlier determination by ROUT method, Q=5%.
   b. Calculate the mean, standard deviation (SD) and %CV of absorbance value of NC and PC after removing outliers.
   c. Calculate the cutoff point from negative control signal by mean absorbance value +1.645 SD.
   d. The titer of each sample is determined by the maximum dilution factor that OD value above the cutoff point.

Flow cytometry analysis procedure

1. The splenocytes harvested from immunized mice were grinded into a single cell suspension from step2-9.
2. The digested spleen will be crushed through a 70 µm nylon mesh cell strainer using the plunger of a 5 mL syringe and transfer to a fresh 50 mL tube, and wash cells through cell strainer using 5 mL of PBS.
3. The cells will be centrifuged at 450 g for 5 min at 4°C.
4. The pellet will be resuspended in 3 mL of ACK (ammonium-chloride-potassium) lysis buffer for lysis of red blood cell.
5. The mixture will be equilibrated at room temperature for 5 min followed by addition of PBS to a final total volume of 6 mL for neutralization.
6. The mixture will be centrifuged at 450 g for 5 min at 4°C, followed by removal of supernatant. The pellet should have a white color (if a red color is observed, repeat the lysis step).
7. The pellet will be re-suspended in 1 mL PBS and filter through 70 µm cell strainer to a new 50 mL tube.
8. The strainer will be washed with PBS to reach a total volume 5 mL.
9. The cell number will be counted and adjusted to a concentration of 1 $x10^7$ cells/mL in PBS.
10. The suspended cells were seeded on 96-well U-bottom plates at density of 2×10^6 cells/well and stimulated with SARS-CoV-2 S1 mixed peptide pools (2 µg/mL, Mabtech) for 18-20 hours. The resulting cells were harvested for surface and intracellular markers staining.
11. Cells were resuspended in Cell Staining Buffer (cat. 420201, Biolegend) and incubated with 2µl anti-CD4 (cat.100405, Biolegend) and anti-CD8 (cat.100705, Biolegend) antibodies for surface staining.
12. Fix cells in 0.5 ml/tube Fixation Buffer (cat. 420801, Biolegend) in the dark for 20 minutes at room temperature.
13. Centrifuge at 450xg for 5 minutes, discard supernatant.
14. Wash cells 1x with Cell Staining Buffer (cat. 420201, Biolegend).
15. Resuspend fixed cells in intracellular staining perm wash buffer (cat. 421002, Biolegend) and centrifuge at 450xg for 20 minutes.
16. Resuspend fixed/permeabilized cells in residual intracellular staining perm wash buffer and add 2µl anti-IFN-γ (cat.505809) and anti-IL-4 (cat.504103) antibodies into CD4 stained cells; add 2µl anti-IFN-γ (cat.505809) and anti-Granzyme B (cat.372203) antibodies into CD8 stained cells.
17. The mixture will be incubated on ice for 15-20 minutes in the dark.
18. Wash cells 2x with Cell Staining Buffer.
19. Perform flow cytometry and collect 2 x 10^5 live single cells per panel.
20. All flow cytometry data was acquired by Navious EX flow cytometer (Beckman Coulter) and analyzed by Kaluza software.

CD4 T helper cells: CD4+/IFN-r, CD4+/IL-4.

CD8 T cells: CD8+/IFN-r, CD8+/Granzyme B.

EILSPOT procedure

1. The splenocytes harvested from immunized mice were grinded into a single cell suspension from step2-9.
2. The digested spleen will be crushed through a 70 μm nylon mesh cell strainer using the plunger of a 5 mL syringe and transfer to a fresh 50 mL tube, and wash cells through cell strainer using 5 mL of PBS.
3. The cells will be centrifuged at 450 g for 5 min at 4°C.
4. The pellet will be resuspended in 3 mL of ACK lysis buffer for lysis of red blood cell.
5. The mixture will be equilibrated at room temperature for 5 min followed by addition of PBS to a final total volume of 6 mL for neutralization.
6. The mixture will be centrifuged at 450 g for 5 min at 4°C, followed by removal of supernatant. The pellet should have a white color (if a red color is observed, repeat the lysis step).
7. The pellet will be re-suspended in 1 mL PBS and filter through 70 μm cell strainer to a new 50 mL tube.
8. The strainer will be washed with PBS to reach a total volume 5 mL.
9. The cell number will be counted and adjusted to a concentration of 1 x10^7 cells/mL in PBS.
10. The suspended cells were seeded in 96-well ELISPOT plates at density of 250,000 cells/well in duplicates. Cells were stimulated with 0.4 μg/well SARS-CoV-2 S1 mixed peptide pools (cat. R046-36291, Mabtech) at 37°C for 18-20 hours.
11. Murine IFN-γ, IL-2, and IL-4 ELISPOT were performed following the kit instructions (Cat. MIF00, M2000 and M4000B, Mabtech).
